# Systematic Multi-Omics Integration (MOI) Approach in Plant Systems Biology

**DOI:** 10.3389/fpls.2020.00944

**Published:** 2020-06-26

**Authors:** Ili Nadhirah Jamil, Juwairiah Remali, Kamalrul Azlan Azizan, Nor Azlan Nor Muhammad, Masanori Arita, Hoe-Han Goh, Wan Mohd Aizat

**Affiliations:** ^1^ Institute of Systems Biology (INBIOSIS), Universiti Kebangsaan Malaysia (UKM), Bangi, Malaysia; ^2^ Bioinformation & DDBJ Center, National Institute of Genetics (NIG), Mishima, Japan; ^3^ Metabolome Informatics Team, RIKEN Center for Sustainable Resource Science, Yokohama, Japan

**Keywords:** bioinformatics, co-expression analysis, correlation, k-means clustering, machine learning, multivariate analysis, pathway mapping, modeling

## Abstract

Across all facets of biology, the rapid progress in high-throughput data generation has enabled us to perform multi-omics systems biology research. Transcriptomics, proteomics, and metabolomics data can answer targeted biological questions regarding the expression of transcripts, proteins, and metabolites, independently, but a systematic multi-omics integration (MOI) can comprehensively assimilate, annotate, and model these large data sets. Previous MOI studies and reviews have detailed its usage and practicality on various organisms including human, animals, microbes, and plants. Plants are especially challenging due to large poorly annotated genomes, multi-organelles, and diverse secondary metabolites. Hence, constructive and methodological guidelines on how to perform MOI for plants are needed, particularly for researchers newly embarking on this topic. In this review, we thoroughly classify multi-omics studies on plants and verify workflows to ensure successful omics integration with accurate data representation. We also propose three levels of MOI, namely element-based (level 1), pathway-based (level 2), and mathematical-based integration (level 3). These MOI levels are described in relation to recent publications and tools, to highlight their practicality and function. The drawbacks and limitations of these MOI are also discussed for future improvement toward more amenable strategies in plant systems biology.

## Introduction

The acquisition of multi-omics data sets has become an integral component of modern molecular biology and biotechnology. This is due to technological advancements, such as the next-generation sequencing technology (Illumina, PacBio, and Nanopore) and mass spectrometry coupled with gas- and liquid chromatography, which offer high-throughput data generation (Fondi and Liò, 2015). The core data sets of systems biology are transcriptomics, proteomics, and metabolomics, providing the expression levels of transcripts, proteins, and metabolites, respectively (Aizat et al., 2018a). The data generated from these platforms can be massive, often without clear connections between them. For instance, it is nearly impossible to manually associate hundred thousands of transcripts to their respective proteins or metabolic pathways. In fact, the bottleneck of omics research is considered as the biological/machine/human resource allocation for data processing and integration (Palsson and Zengler, 2010). What we need is a well-defined methodological scheme for multi-omics integration (MOI) to extract, combine, and critically associate different data sets to allow researchers to decipher the seemingly complex biological results at hand (Fondi and Liò, 2015; Hughes, 2015; Wang et al., 2018).

MOI approach has been extensively studied and reviewed in studies on human (Chen and Chen, 2019; Cho et al., 2019; Shetty et al., 2019), animals (García-Sevillano et al., 2014), microbes (Denman et al., 2018; Gutleben et al., 2018; Wang et al., 2019), and their combinations (Wanichthanarak et al., 2015; Cavill et al., 2016; Pinu et al., 2019). In comparison, MOI in plants has been more difficult due to their metabolic diversity, poorly annotated large genomes (particularly for non-model species), and the presence of numerous symbionts with complex interaction networks. Several comprehensive reviews are available specifically on plant MOI (Fukushima et al., 2009; Fukushima et al., 2014; Rajasundaram and Selbig, 2016; Rai et al., 2017; Rai et al., 2019) and its practical usage in green systems biology, precision plant breeding, and other biotechnological applications (Weckwerth, 2011; Weckwerth, 2019; Weckwerth et al., 2020). However, the advancement of high-throughput technologies and large omics data sets leading to big data biology can be overwhelming, and perhaps an “Achilles' heel” for inexperience researchers. Omics data from poorly characterized species are often feed into software without proper manual curation and oblivious of the limitation of each technology, which could result in incorrect interpretations. Further, there are also a large collection of software platforms, statistical rigor, and modeling (Weckwerth, 2011; Pinu et al., 2019) which must be selected appropriately by users, yet these can be viewed as extraneous to untrained researchers. Hence, suitable methodological workflow for MOI must be identified to ensure accurate large-scale data analysis and representation. Previously, the different levels of MOI have been summarized as “conceptual,” “statistical,” and “model-based” integration (Cavill et al., 2016; Rai et al., 2017); instances of such integration have been detailed elsewhere (de Oliveira Dal'Molin and Nielsen, 2013; de Oliveira Dal'Molin and Nielsen, 2018; Seaver et al., 2018). Let us start from a critical review on the previous three-level classification.

The “conceptual” integration refers to multiple omics data sets being analyzed separately and are matched without further statistical analysis. Even though this approach can produce valuable insights, it may miss reproducible associations when multiple omics data sets are analyzed together (Cavill et al., 2016; Rai et al., 2017). We therefore argue that the conceptual integration is an arbitrary connection without proper analysis and should not be considered a part of MOI approach. Instead, we re-classify the “statistical” integration where statistical associations are sought between elements from different data sets (Cavill et al., 2016; Rai et al., 2017). The effective use of prior knowledge is separated as the pathway-based integration from unbiased, element-based integration. Finally, “model-based” integration is also re-classified so that qualitative reconstruction of biological pathways or systematic regulatory pathways is separated from their quantitative, mathematical evaluation to generate working models for hypothesis testing (Thiele and Palsson, 2010; Rai et al., 2017).

**Figure 1:**
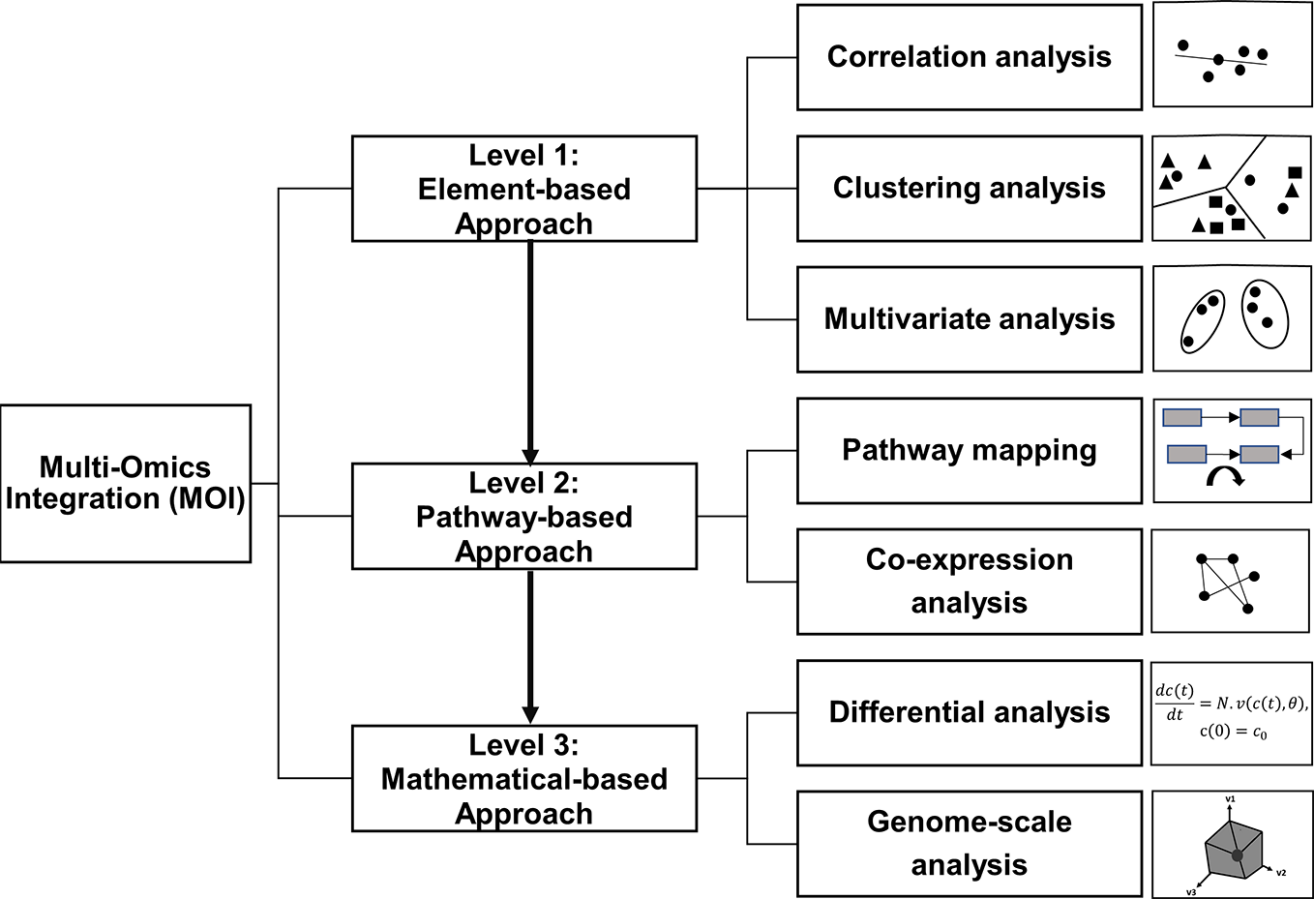
Current approaches in multi-omics integration (MOI) of plant systems biology. This MOI strategy is classified into three main levels with increasing degrees of complexity.

Thus, we re-define the MOI workflow into three main integration levels (levels 1 to 3) with increasing complexity (**Figure 1**). Level 1 is the unbiased, “element-based” integration with three subclasses: correlation, clustering, and multivariate analyses. Level 2 is the knowledge-based “pathway” integration, which includes co-expression and mapping-based approaches. Finally, level 3 is the “mathematical” integration with two subclasses, namely, differential and genome-scale analyses. The three levels are discussed in relation to recent omics reports from the year 2014 to 2020 (**Table 1**) gathered from comprehensive literature searches, including Web of Science, Scopus, and Google Scholar databases providing an updated comprehensive overview of MOI applications in various plant systems. Furthermore, this review focuses on expression-based omics from transcriptomics, proteomics, and metabolomics to further clarify strategies taken to integrate such large-scale expression data (transcript, protein, and metabolite).

**Table 1:** Summary of recent publications and their tools and methods used in the three different levels of multi-omics integration (MOI).

Integration type	Organisms	Common name	Study purpose	Transcriptomics	Proteomics	Metabolomics	Method/Software	Reference
**Level 1 (Element-based approach)**								
Correlation	*Arabidopsis thaliana*	Thale cress	Seed germination and seedling growth	Yes	N/A	Yes	Pearson	Silva et al., 2017
	*Gossypium hirsutum*	Upland cotton	Salt tolerance	Yes	Yes	N/A	Pearson	Peng et al., 2018
	*Persicaria minor*	Kesum	MeJA treatment	Yes	Yes	N/A	Pearson	Aizat et al., 2018b
	*Solanum lycopersicum*	Tomato	Fruit ripening	Yes	Yes	N/A	Fisher transformation	Mata et al., 2018
	*Zizipus jujuba*	Jujube red dates (leaf)	Bacterial (phytoplasm) infection	Yes	Yes	N/A	Spearman	Ye et al., 2017
	*Ginkgo biloba*	Ginkgo	Age (young and mature leaf)	Yes	N/A	Yes	Pearson	Guo et al., 2020
Clustering	*Solanum lycopersicum*	Tomato	Pollen development	Yes	Yes	N/A	*k-*means (MEV)	Keller and Simm, 2018
	*Solanum tuberosum*	Potato	Network construction for traits prediction	Yes	Yes	Yes	Random Forest regression	Acharjee et al., 2016
	*Theobroma cacao*	Cacao	Seed development	N/A	Yes	Yes	*k*-means	Wang et al., 2016
	*Vitis vinifera*	Grapevine	Fruit development and ripening	N/A	Yes	Yes	*k*-means	Wang et al., 2017
Multivariate	*Persea americana*	Hass avocado	Heat shock treatment	N/A	Yes	Yes	DIABLO (mixOmics in R)	Uarrota et al., 2019
	*Populus* sp.	Aspen	Wood formation	Yes	Yes	Yes	OnPLS	Obudulu et al., 2018
	*Zea mays*	Maize	Herbicide tolerance	N/A	Yes	Yes	MCIA in R	Mesnage et al., 2016
	*Zea mays*	Maize	Lipid biosynthesis	Yes	N/A	Yes	GFLASSO	De Abreu E Lima et al., 2018
**Level 2 (Pathway-based approach)**								
Pathway mapping	*Arabidopsis thaliana*	Thale cress	Plastidial retrograde signaling	Yes	Yes	Yes	PathVisio	Bjornson et al., 2017
	*Glycine max*	Soybean	Fungal infection	Yes	N/A	Yes	KEGG	Zhu et al., 2018
	*Glycine max*	Soybean	Nematode infection	Yes	N/A	Yes	KEGG	Kang et al., 2018
	*Quercus ilex*	Holm Oak	Pathway reconstruction using multi-tissues	Yes	Yes	Yes	MapMan	López-Hidalgo et al., 2018
	*Persicaria minor*	Kesum	MeJA response	Yes	N/A	Yes	KEGG	Rahnamaie-Tajadod et al., 2017; Rahnamaie-Tajadod et al., 2019
	*Santalum album*	Sandalwood	Santalol (sesquiterpene) biosynthesis	Yes	Yes	N/A	KEGG	Mahesh et al., 2018
Co-expression	*Citrus sinensis*	Sweet orange	Fungal tolerance	Yes	N/A	Yes	Cytoscape	He et al., 2018
	*Vitis vinifera*	White grapes	Water deficit treatment	Yes	N/A	Yes	WGCNA, Cytoscape	Savoi et al., 2016; Savoi et al., 2017
	*Zea mays*	Maize	Maize development	Yes	Yes	N/A	WGCNA	Walley et al., 2016
	*Zea mays*	Maize	Maize development	Yes	Yes	N/A	Weighted interaction network	Jiang et al., 2019
**Level 3 (Mathematical-based approach)**								
Differential	*Arabidopsis thaliana*	Thale cress	Cold stress	Yes	N/A	Yes (in silico predicted)	Metabolite-Centric Reporter Pathway Analysis (RPA^m^)	Koç et al., 2018
	*Solanum lycopersicum*	Tomato	Modeling protein changes during ripening	Yes	Yes	N/A	ODE	Belouah et al., 2019
	*Populus trichocarpa*	Poplar	Lignin biosynthesis	Yes	Yes	Yes	ODE	Wang et al., 2018a
	*Vitis vinifera*	Grape	Anthocyanin biosynthesis	N/A	Yes (enzymatic assays)	Yes	FBA	Soubeyrand et al., 2018
Genome-scale	*Arabidopsis thaliana*	Thale cress	Growth hormone flux *via* root	Yes	Yes	N/A	PlantSEED	Scheunemann et al., 2018
	*Brassica napus*	Rapeseed	Seed development	Yes	N/A	Yes	bna572+ database and FVA	Hay et al., 2014
	*Glycine max*	Soybean	Development of mature seed	Yes	N/A	Yes	PMR and MetNetDB	Li et al., 2015
	*Setaria italica*	Foxtail millet	C4 plant metabolism	Yes	Yes	Yes	C4GEM	de Oliveira Dal'Molin et al., 2016
	*Zea mays*	Maize	Leaf development	Yes	N/A	Yes	CornCyc 4.0, MetaFlux	Bogart and Myers, 2016

MeJA, methyl jasmonate; N/A, not applicable/available; DIABLO, Data Integration Analysis for Biomarker discovery using a Latent component method for Omics studies; FBA, flux balance analysis; FVA, flux variability analysis; GFLASSO, graph-guided fused least absolute shrinkage and selection operator; KEGG, Kyoto Encyclopedia of Genes and Genomes; MCIA, multiple co-inertia analysis; MEV, Multiple Experiment Viewer; OnPLS, orthogonal projections to latent structures; ODE, ordinary differential equation; PMR, Plant/Eukaryotic and Microbial Metabolomics Systems Resource; WGCNA, Weighted Gene Co-expression Network Analysis.

## Level 1 MOI: Element-Based Approach

### Correlation Analysis

The first level of MOI is an element-based integration approach specifically using correlation analysis (**Figure 1**). The advantage of this integration is its simplicity and intuitiveness. The standard approach is correlative association between two or more different omics data sets (i.e., transcriptomics, proteomics, and metabolomics data sets). Such analysis is performed using Pearson's (Benesty et al., 2009) and Spearman's correlation coefficients (Myers and Sirois, 2004), which assess linear and ranked relationships, respectively. Other studies also analyzed their omics data sets using Fisher's transformation to transform skewed data sets to normally distributed data for calculating corresponding correlation coefficients (Mata et al., 2018). In general, significant positive or negative coefficient suggests strong direct or inverse relationship between data sets, respectively.

Correlation-based MOI has been performed between transcripts and their cognate proteins. Such analysis is straightforward, assuming that differential expression of transcripts will also be observed at their translational (protein) level. However, this is often not the case, most studies reported weak correlations (different patterns) between transcript-protein levels. For instance, salt treatment on salt-tolerant Earlistaple 7 and salt-sensitive Nan Dan Ba Di Da Hua cotton revealed scarce correlation (r=0.03) between transcript and corresponding protein patterns, regardless of genotypic background (Peng et al., 2018). Another example includes methyl jasmonate (MeJA) stress hormone treatment on *Persicaria minor* Huds. herbal plants, with poor overall proteome-transcriptome correlation (r=0.341) (Aizat et al., 2018b). Similarly, transcripts and proteins related to ethylene pathway (ethylene receptors [ETRs] and downstream signaling proteins, constitutive triple response-like proteins [CTRs], and ethylene insensitive 2 [EIN2]) were not well correlated during the ripening process of tomato (*Solanum lycopersicum*) (Mata et al., 2018). This suggests the existence of post-transcriptional and post-translational regulation (such as proteasomal degradation) for the majority components of stress and ripening pathways. Despite transcriptome can be weakly correlated to proteome, it serves as an excellent database for protein identification in proteomics informed by transcriptomics approach for non-model plants (Aizat et al., 2018b; Wan Zakaria et al., 2019) as well as studying allele-specific expression (van Wesemael et al., 2018).

On the other hand, an interesting emerging pattern arises when transcript-protein is compared between specific protein groups. For example, significantly upregulated proteins were positively correlated with their cognate transcripts in the stress response of various plants (Ye et al., 2017; Aizat et al., 2018b). Specifically, proteins related to defense such as proteases and peroxidases in MeJA-treated *P. minor* (Aizat et al., 2018b) and secondary metabolite biosynthesis such as flavonoid in phytoplasma-infected *Ziziphus jujuba* Mill. leaf (Ye et al., 2017) were upregulated coherently with their transcripts. This may suggest the concerted molecular upregulation of defense-related proteins to overcome stress signals and infection. Meanwhile, proteins related to growth such as photosynthetic and structural proteins were significantly suppressed in these studies, perhaps as a response toward the stress signal to conserve energy and recycling molecular resources (Ye et al., 2017; Aizat et al., 2018b). Interestingly, such downregulation was mainly observed at the protein level, but not the transcript level (Aizat et al., 2018b), perhaps as a mechanism to quickly resume protein synthesis, when the stress is relieved. However, we could not rule out the possibility that changes at both transcript and protein levels are not simultaneous. Even when sampling of both is done at the same time, the translational and post-translational degradation and modification rates may differ among proteins. However, this is often unpredictable from the genome sequences alone (Weckwerth, 2019; Weckwerth et al., 2020), convoluting meaningful and direct interpretation between expression data.

While comparisons between transcripts and corresponding proteins are generally performed in multi-omics studies, correlation of these two with metabolites are relatively fewer. Perhaps, one such recent example is the transcriptomics and metabolomics investigation of *Ginkgo biloba* during leaf maturity process (Guo et al., 2020). Correlation analysis was performed in this study between all differentially expressed transcript (DET) and metabolites (DEM), however with no regard for their biochemical pathway relationships. While this study may be interested only in the pattern consistency between DET and DEM, the corresponding biochemical pathway should be considered before such correlation is performed. Importantly, metabolites should be classified as either being substrates or products of certain enzymatic pathways to be accurately correlated with their corresponding transcripts/proteins. This has been performed by Silva et al. (2017) for transcripts and associated metabolites to elucidate primary metabolism in Arabidopsis seed germination and growth.

### Clustering Analysis

Clustering analysis allows grouping of omics data sets with similar attribute such as expression levels to deduce underlying associations and patterns. There are two main approaches in clustering, either hierarchical such as HCA (hierarchical cluster analysis) or non-hierarchical methods. However, the latter approach (non-hierarchical) is more applicable in the integration of multiple omics especially using machine learning algorithms, such as the *k-*means clustering and random forest (Ma et al., 2014; Silva et al., 2019). *k*-means clustering groups available data points (in this case from the omics expression data) such that clear, distinctive groupings emerge to differentiate expression patterns. Meanwhile, random forest classifies a group of genes/proteins/metabolites based on prior training data sets (from omics experiments) to associate them to a particular characteristic/trait of interest (Ma et al., 2014). These techniques have been used widely in plant multi-omics research.

For instance, Keller and Simm (2018) reported two modes of protein translation when comparing transcriptome and proteome of tomato pollen development under either control or heat stress condition. The study employed the *k*-means clustering approach (**Table 1**), which clustered expressed transcripts and proteins to different clusters according to developmental stages. This has revealed the underlying mechanism for protein translation; one that significantly correlated between transcript-protein pair at one particular stage (direct translation) or if certain proteins were only differentially expressed in the next stage after their corresponding DET at one stage (delayed translation). The latter phenomenon may explain the weak correlation between transcript-protein pairs at certain stages of pollen development, primarily those proteins related to carbohydrate and energy metabolism. Furthermore, upon heat stress, heat-shock proteins were regulated mostly at the translational level (synthesis and degradation) rather than transcription, suggesting immediate plant response toward stresses (Keller and Simm, 2018).

In addition, *k*-means clustering approach has also been used to integrate proteomics and metabolomics (**Table 1**) from developing cacao seeds (Wang et al., 2016) and grape fruits (Wang et al., 2017). Such integration successfully identified stage-specific clusters, whereby secondary metabolites such as flavonoids were found concomitantly increased with the upregulation of corresponding biosynthetic enzymes (Wang et al., 2016; Wang et al., 2017). Interestingly, Granger causality network analysis performed by Wang et al. (2017) on co-regulated clusters further revealed significant time-shift correlation between protein and metabolite pairs in grapes. This suggests that protein abundance may be directly responsible for metabolic modulation during fruit development and ripening (Wang et al., 2017) highlighting the importance of systematic MOI in elucidating key regulatory elements in plants.

In another study by Acharjee et al. (2016), a random forest approach was utilized to cluster and correlate transcriptomics, metabolomics, and proteomics data sets against certain potato tuber phenotypic traits (flesh color, shape, starch gelatinization, and discoloration after peeling). Interestingly, this study revealed that the different omics was associated strongly with the different tuber traits (Acharjee et al., 2016). For example, traits related to color were more likely to be correlated to metabolite data (such as carotenoids) whereas tuber shape was influenced strongly by transcripts related to size. This implies that certain omics are more suited to reveal the underlying mechanism of a certain phenotypic or experimental condition. Hence, it is important to choose the most suitable omics platform for any investigation, especially those related to phenotypic changes for relatable and descriptive results.

### Multivariate Analysis

Multivariate analysis can handle more complex omics data sets, while allowing greater flexibility in experimental design and metadata analysis (Rai et al., 2017). This approach enables the user to predict different aspects or trends of data sets, including the discovery of variance or covariance associations (Meng et al., 2014) as well as investigating the dynamic relationships and topological networks between transcript/protein/metabolite elements (Weckwerth, 2019). Among the most common multivariate techniques are principal component analysis (PCA), partial least squares (PLS) and orthogonal projection to latent structures discriminant analysis (OPLS-DA) (Mamat et al., 2018; Mazlan et al., 2018; Reinke et al., 2018). Selecting different multivariate techniques, optimal parameters, and model validation can be overwhelming for new users, and hence several reading materials on this topic provide excellent learning resources (Tabachnick et al., 2007; Meng et al., 2014; Saccenti and Timmerman, 2016).

Recently, Obudulu et al. (2018) performed OnPLS (multiple-block orthogonal projections to latent structures), an extension of the OPLS technique, to integrate transcriptomics, proteomics, and metabolomics of poplar transgenic plants lacking *PttSCAMP3* (*Populus tremula* x *tremuloides* Secretory Carrier-Associated Membrane Protein3) gene, potentially important for wood development. Evidently, several biomarkers related to the wood formation and secondary cell wall components have been successfully documented using this approach. Other forms of multivariate analyses, such as MCIA (multiple co-inertia analysis) and GFLASSO (graph-guided fused least absolute shrinkage and selection operator) have also been applied in multi-omics plant studies. For instance, MCIA was used to integrate metabolome and proteome of a near-isogenic maize line (control) and its transgenic counterpart (glyphosate-tolerant maize, NK603) (Mesnage et al., 2016). The study successfully identified metabolic differences between the two, in particular, sugar metabolism and polyamine biosynthesis (Mesnage et al., 2016). Another study in maize further illustrates the use of multivariate analysis such as GFLASSO to integrate transcriptome and metabolome in deciphering its lipid biosynthesis (De Abreu E Lima et al., 2018).

Furthermore, the integration of proteomics and metabolomics data of early and middle season Hass avocado using multivariate dimension reduction discriminant analysis method, DIABLO (Data Integration Analysis for Biomarker discovery using a Latent component method for Omics studies) led to the identification of correlated discriminatory variables that linked the effect of heat treatment to ripening homogeneity (Uarrota et al., 2019). Both of the omics data sets revealed noticeable differences between early and middle season avocados after 1-day heat treatment. Positive correlation was observed between proteins and metabolites for treated middle season fruit particularly those involved in nitrogen recycling and protein degradation. This is perhaps due to carbon starvation induced by the lower rate of glycolysis upon such treatment. This possibly stimulated protein degradation to supply amino acids as substrates for the TCA cycle. Furthermore, the heat treatment induced the accumulation of sucrose, galactinol, and stress-related enzymes which may contribute to the coherent ripening process in avocado (Uarrota et al., 2019). These studies suggest that MOI using multivariate analysis is an efficient strategy to classify and associate various omics to reveal important findings and trends.

While this level 1 MOI proves to be useful in plant omics data integration, these omics data sets are often analyzed numerically, without emphasis on interacting partners or co-expressed molecules as well as underlying biological pathways (or at least this must be done manually at users' discretion). This may impede further understanding of interrelated molecular regulation between different omics and their biological significance in plants upon certain treatments or conditions. Hence, level 2 MOI, which is a pathway-based integration, will be required for the next degree of omics data integration.

## Level 2 MOI: Pathway-Based Approach

### Pathway Mapping

Pathway mapping is aimed to map omics data sets, either transcriptome, proteome or metabolome to existing metabolic pathway database. One prominent database used for plant metabolic pathway reference is Kyoto Encyclopedia of Genes and Genomes (KEGG; https://www.genome.jp/kegg/), but other more organism-specific databases such as AraCyc for Arabidopsis (https://www.arabidopsis.org/biocyc/), CitrusCyc for citrus (https://www.citrusgenomedb.org/node/1136703), and SolCyc for *Solanaceae* species (https://solgenomics.net/tools/solcyc/index.pl) (Foerster et al., 2018) do exist. These databases hold key information for pathway annotation and the basis of a number of software available for MOI at the pathway level (**Table 2**).

**Table 2:** Summary of software tools and web applications for MOI in plant system (modified from Pinu et al., 2019).

Software	Supported omics platform	License type (Vendor)	Functionality	Supported MOI level	Website	Reference
BioCyc/MetaCyc	Genomics Transcriptomics Proteomics Metabolomics	Open source (SRI International)	Metabolic pathway prediction in sequenced genome	2, 3	https://biocyc.org https://metacyc.org	Caspi et al., 2015
C4GEM	Transcriptomics Proteomics Metabolomics	Open source (within ModelSEED)	Metabolic modeling	3	https://modelseed.org/	de Oliveira Dal'Molin et al., 2010
COBRA (Constraint-based reconstruction and analysis)	Transcriptomics Proteomics Metabolomics Fluxomics	Open source (within MATLAB)	Genome-scale modeling	3	https://opencobra.github.io/cobratoolbox	Orth et al., 2010
COVAIN (Covariance inverse)	Transcriptomics Proteomics Metabolomics	Open source (within MATLAB)	Multivariate statistics Granger time-series analysis Network topology Pathway mapping	1, 2, 3	http://www.univie.ac.at/mosys/software.html	Sun and Weckwerth, 2012
GIM^3^E	Transcriptomics Metabolomics	Open source (within COBRApy)	Flux metabolites prediction	3	https://github.com/brianjamesschmidt/gim3e	Schmidt et al., 2013
IMPaLA (Integrated molecular pathway level analysis)	Transcriptomics Proteomics Metabolomics	Open source	Enrichment analysis Pathway analysis	2	http://impala.molgen.mpg.de	Kamburov et al., 2011
KaPPA-View4	Transcriptomics Metabolomics	Open source (Kazusa DNA Research Institute)	Pathway mapping Data visualization	2	http://kpv.kazusa.or.jp/kpv4/	Sakurai et al., 2010
KBCommons (Knowledge Base Commons)	Phenomics Epigenomics Genomics Transcriptomics Proteomics Metabolomics	Open source (KBCommons)	Universal framework for data management and retrieval	2	https://kbcommons.org	Zeng et al., 2019
MapMan4	Transcriptomics Proteomics Metabolomics	Open source	Enrichment analysis Visualization of data expression	2	https://mapman.gabipd.org	Schwacke et al., 2019
mixOmics	Metagenomics Transcriptomics Proteomics Metabolomics	Open source (within R)	Data integration Similarity relationship	1	http://www.mixOmics.org	Rohart et al., 2017
Omicade4	Transcriptomics Proteomics Metabolomics	Open source (within R)	Analyze co-relationship between data sets	1	http://bioconductor.org/packages/release/bioc/html/omicade4.html	Meng et al., 2014
OmicsAnalyzer	Transcriptomics Proteomics Metabolomics Fluxomics	Open source (within Cytoscape)	Network mapping	1, 2	https://apps.cytoscape.org/apps/omicsanalyzer	Xia et al., 2010
Omickriging	Transcriptomics Proteomics Metabolomics Fluxomics	Open source (within R)	Data integration and visualization	1	https://github.com/hakyimlab/OmicKriging	Wheeler et al., 2014
OmicsPLS	Metagenomics Transcriptomics Proteomics Metabolomics	Open source (within R)	Data integration and statistical analysis	1	https://github.com/selbouhaddani/OmicsPLS	Bouhaddani et al., 2018
Paintomics3	ranscriptomics Proteomics Metabolomics	Open source	Data integration and visualization Pathway analysis and interaction	2	http://www.paintomics.org	Hernández-de-Diego et al., 2018
PathView	Transcriptomic Metabolomics	Open source	Data integration and visualization	2	https://pathview.uncc.edu/	Luo et al., 2017
PathVisio 3	Transcriptomics Proteomics Metabolomics	Open source	Pathway editor and data visualization Pathway analysis	2	https://pathvisio.github.io/	Kutmon et al., 2015
PlantSEED	Genomics Transcriptomic Metabolomics Fluxomics	Open source (within ModelSEED)	Metabolic reconstruction	3	https://modelseed.org/	Seaver et al., 2018
Regulomics	Epigenomics Transcriptomics	Open source	Functional characterization of data Retrieve upstream regulators Data mining	2	http://bioinfo.sibs.ac.cn/plant-regulomics/	Ran et al., 2020
VANTED (Visualization and Analysis of Network)	Metagenomics Transcriptomics Proteomics Metabolomics	Open source (Java)	Metabolic mappings Correlation networks analysis	2	https://www.cls.uni-konstanz.de/software/vanted/	Hartmann and Jozefowicz, 2018

Some of these software tools include MapMan and PathVisio, which have been used to study and integrate multi-omics data sets from plants (**Tables 1** and **2**). For example, the integration of transcriptome, proteome, and metabolome using MapMan software allows the Holm oak (*Quercus ilex*) metabolic pathways to be visualized and reconstructed (López-Hidalgo et al., 2018). Evidently, these omics data sets were successfully mapped into 123 out of 127 available KEGG pathways, and pathways such as citrate cycle were shown to be highly enriched in this study (López-Hidalgo et al., 2018). Besides that, an integration between transcriptomics, proteomics, and metabolomics was also performed using PathVisio in the study of Arabidopsis signaling mutant plants (Bjornson et al., 2017). This study revealed that the mutant with a high level of methylerythritol cyclodiphosphate compound perturbed numerous stress-response signaling pathways, including biosynthesis of jasmonate and salicylate (Bjornson et al., 2017). This shows the practicality of these software tools for elucidating and revealing the inherent modulation of certain biochemical pathways in plant multi-omics studies.

Other software such as IMPaLA (integrated molecular pathway level analysis) (Kamburov et al., 2011), Paintomics (García-Alcalde et al., 2010), and InCroMAP (integrated analysis of cross-platform microarray and pathway data) (Eichner et al., 2014) are also available (**Table 2**) but their applications in plant MOI are limited. The principles of integrating multi-omics vary between the tools used. For example, PathVisio, Paintomics, and InCroMAP produce a joint-pathway *P*-value by totalling the number of differentially expressed components in each omics prior to combining them with the total number of measured data sets (Cavill et al., 2016). Other tools such as IMPaLA uses Fisher's method when combining *P*-values from multiple tests of the same hypothesis (Kamburov et al., 2011; Cavill et al., 2016). In the future, more software and databases for specific plant species are expected to be developed as metabolic pathways in different plants can be unique and diverse.

On the other hand, it is also possible to reconstruct integrated biochemical pathways manually without assistance from any of these software tools. Canonical pathways from the available databases such as KEGG can be reconstructed specifically for any organism of interest, based on its annotated enzymes and/or metabolites. Although this method is labor intensive, it proves to be useful in elucidating targeted pathways. For instance, the transcriptomics and metabolomics studies of soybean infected with *Phytophthora sojae* fungus (Zhu et al., 2018) or cyst nematode (Kang et al., 2018) revealed transcriptional and metabolic modulation toward isoflavonoid and phenylpropanoid biosynthetic pathways, respectively based on KEGG database. These studies highlight the complex interplay and response between plants and interacting rhizosphere. Another study using KEGG pathway as a reference was reported for *Persicaria minor* elicited with MeJA phytohormone (Rahnamaie-Tajadod et al., 2017; Rahnamaie-Tajadod et al., 2019). These reports integrated transcriptome and metabolome data sets into manually reconstructed terpenoid and sesquiterpenoid biosynthetic pathways specifically for this non-model plant (Rahnamaie-Tajadod et al., 2017; Rahnamaie-Tajadod et al., 2019). Additionally, santalol (sesquiterpene) biosynthesis from sandalwood (*Santalum album*) was also successfully reconstructed using multi-omics annotation to available KEGG database (Mahesh et al., 2018). These studies suggest that manual pathway reconstruction using available pathway databases is possible for pathway-based MOI, albeit time-consuming.

However, it is to be noted that performing annotation for pathway mapping across different species are often tricky. It is a common practice to BLAST sequences from non-model plants against model plants (e.g., Arabidopsis) and accepting the best hit from the BLAST results. Inexperience researchers tend to accept this annotation without considering if the conserved domains or functions are significantly recognized. The lack of confidence in cross-species annotation may restrict the reliability of generated biochemical pathway relationships (Weißenborn and Walther, 2017) and other downstream analyses such as protein-protein interaction analysis (Mika and Rost, 2006). Hence, users must critically examine and curate the annotation and pathway mapping results with further experimental validations through targeted functional analysis to avoid misrepresentation or erroneous conclusion.

### Co-expression Analysis

Co-expression analysis heavily relies on statistical correlations between different omics data sets, as discussed earlier in the first level of MOI, to assess the strength of relationships between expressed molecules (Voigt and Almaas, 2019). Such relationships are then transformed into a weighted network and can be visualized using a few tools including Weighted Gene Co-expression Network Analysis (WGCNA) in R program or Cytoscape tool. This strategy has revealed important clusters, modules, and hubs for biological insights pertaining to specific pathways or regulatory molecules in various plant studies.

For instance, WGCNA approach followed by Cytoscape visualization was used to elucidate the transcriptome and metabolome relationship in white grapes (*Vitis vinifera* L.) during prolonged drought (Savoi et al., 2016; Savoi et al., 2017). Eleven modules with different co-expression patterns were reported in this study, which emphasized on the regulatory network of transcription factors and secondary metabolic pathways. Among others, auxin and abscisic acid (ABA) signaling have been shown to be key regulatory components during the water deficit stress of which these were well connected to the modulation of secondary metabolites such as phenylpropanoids and flavonoids (Savoi et al., 2017). Another study in maize development also utilized WGCNA for transcriptomics, proteomics, and phosphoproteomics integration (Walley et al., 2016). They have developed an expression atlas that encompassed 23 different maize tissues from vegetative to reproductive stages and further analyzed their relationship through the weighted networks. Interestingly, highly connected network hubs were different for co-expressed transcripts and proteins generated from the transcriptome and proteome data sets, respectively. This could be due to a smaller percentage of proteins (46%) were detected from the full transcriptome list, and hence may affect the co-expression and network analysis within that data sets. Further analysis of these data sets was performed by Jiang et al. (2019). By integrating the different weighted networks from respective omics into a fused network, a consensus network supported by the evidence from all the different omics studies was successfully generated. This exercise further illuminates the integral roles of various transcription factors in the molecular regulation of maize development (Jiang et al., 2019).

Another form of co-expression analysis is through the integration with pathway databases. This was reported for an integrated transcriptomics and metabolomics study in orange, *Citrus sinensis* (He et al., 2018). This study compared “Newhall” navel orange, a spontaneous mutant variety that is highly resistant to a broad range of fungi infection with its wildtype. A species-specific database (CitrusCyc2.0) was employed to identify pathways with the highest correlation between transcriptional and the metabolic data. Networks for upregulated and downregulated elements (transcripts and metabolites) were generated using Cytoscape to contrast the mutant and wildtype which suggest the former variety was tolerant to fungi attack through fatty acid compositional changes and subsequently the induction of JA-mediated response for defense (He et al., 2018). Another study in maize development utilized MapMan functional annotation to decipher enriched pathways for their highly connected co-expressed hubs (Walley et al., 2016). Thus, these different studies show that the co-expression network analysis integrated with pathway databases is a powerful tool to obtain insights into the plant development and stress response, allowing omics data sets to be arranged in highly connected modules and hubs for further investigation.

MOI using software tools, either for pathway mapping or co-expression analyses, undeniably simplify the integration task, especially to find relationships between omics data sets, metabolic pathways, and their regulation. However, such pathway templates are often static, unamenable toward changes in experimental parameters and perturbation, as well as may not be organism-specific. Therefore, there is a need to accurately predict metabolic changes (perturbation) upon certain pre-set conditions or treatments in specific species of interest, which will be detailed in level 3 MOI, mathematical-based integration.

## Level 3 MOI: Mathematical-Based Approach

### Differential Analysis

The mathematical-based MOI poses as the most complex integration of all, and this approach requires extensive omics data coverage and well-characterized plants. One of the most basic aim in the mathematical-based approach is to develop a well-defined differential equation and modeling for a systems-level understanding. Such analysis consists of four main steps: identification of systems components, determination of systems regulation and topology, the development of appropriate mathematical equations, and lastly, parameter selection and optimization (Voit, 2017).

Differential analysis has been applied to various plant and fruit studies (Koç et al., 2018; Wang et al., 2018; Belouah et al., 2019) and can be divided into either non-targeted or targeted pathway studies. One recent example of the former (non-targeted pathway) approach is the development of differential equation for protein density during tomato ripening (Belouah et al., 2019). This is performed by integrating transcriptomics and proteomics data sets of nine tomato developmental stages using ordinary differential equations (ODE) to obtain rate constants for translation (*k_t_*) and degradation (*k_d_*). The result suggests that the equation reliably predicts the expression of nearly 50% of 2,400 transcript-protein pairs from the study and that the protein level was regulated strongly by the translation rate rather than degradation (Belouah et al., 2019).

For a targeted pathway approach, differential analysis can be used to model a specific pathway for its metabolic flux and dynamics. For instance, lignin biosynthesis in poplar (*Populus trichocarpa*) was successfully modelled using the ODE approach (Wang et al., 2018). This study first generated RNAi mutant plants for 21 target genes of the monolignol pathway before transcriptomics and targeted proteomics were performed on these transgenics. Transcript-protein equation was developed to model the effect of the gene silencing and ODE was used to generate mass-balance kinetics to predict metabolite levels and fluxes. Such a model consistently predicts the effect of gene perturbation in improving lignin content and wood properties and hence will be of interest to the breeding program of this valuable tree species. Additionally, another mathematical analysis study in targeted pathways was performed on Arabidopsis acclimatized to cold stress (Koç et al., 2018). Microarray (transcriptomics) data sets were obtained for four periods of cold conditions before the expression data was mapped to available metabolic pathways from AraCyc and KEGG. Using Reporter Metabolic Centric Algorithm in Matlab, DEGs were linked to corresponding metabolites before metabolite and pathway scores (called reporter metabolite and reporter pathway, respectively) were calculated. Tripartite network model encompassing gene, metabolite and pathways were built which revealed stress modulated pathways related to carbon, redox, and signal metabolisms upon the cold treatment (Koç et al., 2018). Furthermore, constraint-based modeling such as flux balance analysis (FBA), usually performed using COBRA (constraint-based reconstruction and analysis) toolbox in Matlab application (Orth et al., 2010) has also been used in this mathematical modeling (**Table 2**). For example, anthocyanin biosynthesis in grapes, *Vitis vinifera* has been successfully modelled using FBA through data generated from metabolomics, proteomics (enzymatic activity), and growth experiment (Soubeyrand et al., 2018). The result further suggests that anthocyanin metabolic flux is strongly induced upon nitrogen deprivation, as a mean of excess energy utilization (Soubeyrand et al., 2018).

These studies show that differential analysis can be useful for MOI in plants either with or without specific target pathways (Voit, 2017). Integration at this level, particularly for targeted pathway may depend upon a complete, well-annotated single metabolic pathway and hence may be applied to both model and non-model organisms alike, provided that sufficient molecular information is available. Various other resources for this mathematical and flux analyses are also available, for instance, E-flux (https://omictools.com/e-flux-tool) and Metabolic Adjustment by Differential Expression (https://omictools.com/made-tool) which have been reviewed comprehensively by Fukushima et al. (2014). Nonetheless, differential analysis also serves as a crucial component for further omics integration in genome-scale analysis (Cavill et al., 2016).

### Genome-Scale Analysis

Previously in differential analysis, the stoichiometric equation is only developed for a specific purpose, such as measuring translation rate or metabolic flux in one isolated system or pathway. Furthermore, this top-down approach relies upon experimental results to construct a functional mathematical model (Fukushima et al., 2014; Voit, 2017). In contrast, a genome-scale modeling (GSM) aims to build the (genome-scale) model first from extensive curation before experimental validation, and hence denoted as a bottom-up approach (Thiele and Palsson, 2010; Voit, 2017; Goh, 2018). This approach aims to complete metabolic pathways at the organism- and cellular-wide levels such that each and every single reaction is considered for a holistic mathematical evaluation (de Oliveira Dal'Molin and Nielsen, 2018). The process of developing genome-scale metabolic reconstructions has been extensively reviewed previously (de Oliveira Dal'Molin and Nielsen, 2018; Seaver et al., 2018) and mainly consists of four large steps: draft reconstruction using annotated genome, pathway refinement using experimental results, network modeling in mathematical format, and lastly, validation and iteration for model accuracy (Thiele and Palsson, 2010).

GSMs for plants and other eukaryotes are significantly more complicated than those for prokaryotes due to their extensive compartmentalization, size, polyploidy as well as numerous and variegated secondary metabolic pathways (Rai et al., 2017; Rai et al., 2019). For plant metabolic reconstruction, a streamline GSM database called PlantSEED (**Table 2**) provides a metabolism-centric resource for annotating metabolic reactions in new plants based on 10 well-annotated plant genomes (Seaver et al., 2018). In general, this database is useful for genome-scale reconstructions particularly for primary metabolism, but manual curation is still required for specific plant secondary metabolism, which is both rich and highly species-dependent (de Oliveira Dal'Molin and Nielsen, 2018).

GSM tool such as PlantSEED facilitates multi-scale analysis allowing researchers to explore complex metabolic processes varying from single cells to multiple tissues at a whole plant level (de Oliveira Dal'Molin et al., 2016). For instance, PlantSEED was used to model the multi-cell root system in Arabidopsis, by supplementing transcriptomics and metabolomics information (Scheunemann et al., 2018). The study managed to predict the metabolic flux of indole-3-acetic acid, a key growth regulator in Arabidopsis roots across tissues (Scheunemann et al., 2018). However, PlantSEED genome-wide metabolic reactions are mostly based on C_3_ plants such as Arabidopsis, but not C_4_ model plants such as maize and foxtail millet, *Setaria italic* (de Oliveira Dal'Molin and Nielsen, 2018). Hence, a C_4_ genome-scale model (C4GEM) was developed for such purpose before transcriptomics, proteomics, and metabolomics data sets were mapped to the reconstructed network for functional analysis (de Oliveira Dal'Molin et al., 2016).

Additionally, GSM has also been reported in beans (*Glycine max*) using tools such as Plant/Eukaryotic and Microbial Metabolomics Systems Resource (PMR, http://metnetweb.gdcb.iastate.edu/PMR/) and MetNetDB (https://omictools.com/metnetdb-tool) (Li et al., 2015). This study successfully integrated transcriptomics and metabolomics data sets to functionalize seed filling metabolic model including starch utilization and fatty acid build-up of this legume. Other plants utilized for its seeds, rapeseed (*Brassica napus*) has also been metabolically reconstructed through its Bna572+ database annotation update and a model was developed using Flux Variability Analysis (FVA), one of the methods in FBA (Hay et al., 2014). Transcriptomics and ^13^C metabolic flux experiments were used to build and validate the model of which higher flux for fatty acid biosynthesis was observed in high oil plant genotype (Hay et al., 2014). Similarly, GSM in maize has also been updated and validated through transcriptomics and biochemical assays for its leaf development model (Bogart and Myers, 2016).

Nonetheless, out of all MOI strategies, only mathematical-based integration can accurately predict changes or perturbation in specific organisms due to the application of extensive database annotation as well as validated models using experimental evidence. This includes metabolic flux analysis using isotopic labelling techniques to quantitatively measure cellular flux state (Allen, 2016). However, this effort can be challenging for plants due to diverse cellular/tissue types and organelle compartmentalization (Allen, 2016). Hence, plant flux studies have often been restricted to homogenous cellular/tissue samples and those with extended metabolic steady states such as seeds (Allen, 2016). Such challenges for validating models from this mathematical-based integration approach or other MOI approaches in plant systems must to be considered before attempting any integrated omics experiments.

## Current Challenges and Outlook

The integrative multi-omics approach can often be hindered by differences in data output, variability in data structure, and even noise between technological platforms (Fabres et al., 2017; Pinu et al., 2019). As different omics uses different platforms, one often overlook the bias outputs resulted from the disproportionate amount of identified molecules (transcripts, proteins, and metabolites). Furthermore, MOI can be problematic for data sets that are irreproducible, only qualitative in nature, containing false positives/negatives, and lacking metadata to explain phenotypic changes (Pinu et al., 2019). These general challenges related to MOI and possible rectifications are detailed in Pinu et al. (2019) and references therein. Available software for different MOI strategies are also listed in **Table 2** to aid researchers in choosing suitable approaches. In this review, we highlight the drawbacks related to specific MOI levels to shed light on their future improvement (**Table 3**).

**Table 3:** The advantages, disadvantages, and future outlook for each level of multi-omics integration (MOI) in plant systems biology.

MOI description	Advantages	Disadvantages	Future outlook
**Level 1 (Element-based approach)**			
Correlation	Simple, direct, and intuitive	Limited scope and insights into the biological significance Certain correlation methods such as Pearson can be biased to outliers	Completing gene and metabolite annotation for specific plant species Alternative correlation methods such as bi-weight mid correlation to reduce the impact of outliers
Clustering	Flexibility in data input and sample size	Requires knowledge in machine learning algorithm/tool	More intuitive tools for non-experts
Multivariate	Flexibility in data input and sample size	Different model selections, parameters, and result interpretation can be complicated	More intuitive tools for non-experts
**Level 2 (Pathway-based approach)**			
Pathway-mapping	Intuitive for biologists Many pathway annotation and enrichment tools are available	Moderate to expert level of programming is required D*e novo* gene-metabolite association is not possible, just rely on available annotated pathways Varied annotation to different identifiers from multiple databases, especially metabolites	Species-specific database for gene and metabolite annotation in molecular pathways
Co-expression	Intuitive for biologists Potential molecular interaction can be suggested	Moderate to expert level of programming is required Inconsistent data formatting for downstream tools Mostly for known interactions between genes, proteins, and metabolites	Streamlining various data formats More experiments in enzymatic reactions and protein interactions conducted
**Level 3 (Mathematical-based approach)**			
Differential	Metabolic flux can be simulated and perturbed	Expert level of programming and mathematics is required Optimal model selection and parameter settings can be subjective and complicated Too many molecular components, sometimes redundant, in biological systems that prevent a complete model representation	New data sets and algorithms for model improvement and validation Develop more effective algorithms within intuitive and user-friendly tools
Genome-scale	A curated and accurate model is generated Metabolic flux can be simulated and perturbed	Expert level of programming and mathematics is required Laborious and time-consuming especially for manual curation Extensive resources such as genetics, physiology, and biochemical reactions are needed Lack of evidence for compartmentalized reactions within organelles or intracellular transporters for plants Secondary metabolic pathway modeling still requires extensive manual curation Protein regulation and post-translational modifications as well as environmental impact on metabolic and phenotypic dynamism are not predictable through genome-scale metabolic reconstruction	New data sets for model validation with organellar-specific information More comprehensive large-scale investigation is integrated within the model including epigenomics, post-translational modification proteomics, and phenomics Effective machine learning algorithms for model optimization

Level 1 element-based MOI, such as correlation, clustering, and multivariate, are relatively direct and intuitive analyses, and hence will continue to be among the initial choices for integrating transcriptomics, proteomics, and metabolomics. However, each of these approaches may have individual weaknesses that must be considered by potential users. For example, correlation analysis can be limited in scope and insights into biological knowledge (Cavill et al., 2016), and certain correlation methods, such as Pearson's may be biased to outliers (Usadel et al., 2009). Therefore, future improvements may focus on completing gene and metabolite annotations for various specific organisms to link these elements to phenotypes for a more insightful match. Additionally, alternative correlation methods such as the bi-weight mid-correlation can be used to reduce outliers' impact on the correlated results (Langfelder and Horvath, 2012). The other two approaches (clustering and multivariate) may not be so simple for most beginners. For instance, clustering requires skills in conducting machine learning whereas multivariate analysis needs thorough understanding in selecting appropriate models and parameters so that the result is comprehensible and accurate. On the other hand, these approaches are flexible in terms of data input and size, and hence well suited for visualization of a wide range of data structures and content (Rai et al., 2017). Hence, future tools should be user-friendly especially for beginners to operate and conduct their multi-omics integration.

The next level of MOI (level 2), pathway-based integration which comprises pathway mapping and co-expression analyses also come with their own perks and benefits. For instance, this type of integration is highly intuitive to biologists with many annotation tools and databases available online (Cavill et al., 2016). Potential molecular interactions can also be suggested in the co-expression analysis (Ma et al., 2014), revealing intricate regulation or potential feedback loop between molecules. However, some of the disadvantages for this MOI include the need for moderate to expert level of programming, especially for tools without user-friendly interfaces such as PathQuant (Therrien-Laperrière et al., 2017). Meanwhile, pathway mapping may suffer from the lack of complete annotated pathways especially for non-model organisms, hindering novel association between gene-metabolite (Wanichthanarak et al., 2015). Furthermore, this integration may have varied identifiers between databases, for instance metabolite nomenclature, further complicates integration process, especially in plants which possess high variability of secondary metabolites (Cavill et al., 2016). As sequencing may no longer be an issue due to affordable sequencing technologies with better throughput, such as SMRT (Single Molecule, Real-Time) Pacific Bioscience and Oxford Nanopore (Weckwerth, 2011; Weckwerth et al., 2020) specific databases for various non-model species can be generated to enrich and supplement the gene annotation. Hence this may provide thorough metabolic pathways for various organisms to allow MOI in pathway mapping to be performed holistically. For co-expression analysis, some of the problems include inconsistent data formatting, e.g., KGML (KEGG Markup Language), BioPax (Biological Pathway Exchange), and SBML (Systems Biology Markup Language) for downstream analysis (Fukushima et al., 2014) and the approach is only suited for known established interaction between genes, proteins, and metabolites (Wanichthanarak et al., 2015). Hence, there must be a significant effort streamlining various data formats for the co-expression analysis (Fukushima et al., 2014) while studies characterizing enzymatic reactions and protein interactions must be further supported to aid the integration at this level.

In level 3 MOI, differential and genome-scale analyses are performed. This allows metabolic flux to be simulated, and its perturbation can be predicted *in silico* (Voit, 2017). However, the models built with these approaches require an expert level of programming and mathematics (**Table 3**). In addition, MOI using differential analysis can have several other drawbacks. For instance, model selection and parameter settings can be very subjective and hence highly variable between users depending on their skills and preference (Voit, 2017). Furthermore, a biological system may contain redundant molecular components, hindering a complete model representation (Voit, 2017). Integration using flux balance analysis (FBA) is limited to certain applications such as the ability determining fluxes at steady state, and is unable to predict metabolite concentrations as it does not use kinetic parameter (Orth et al., 2010; Rakwal et al., 2017; Pinu et al., 2019). As construction of FBA does not account for regulatory effects such as enzyme activation, the prediction by this model may lack accuracy except in its modified forms (Orth et al., 2010). Therefore, future efforts may develop a more effective algorithm, yet user-friendly for most biologists (Fukushima et al., 2014). On the other hand, GSM requires a great deal of time and effort to manually curate a complete and thorough model. As such, molecular resources at the genetic level, physiology, and biochemical reactions are paramount for a working GSM (Thiele and Palsson, 2010; Cavill et al., 2016; Rai et al., 2017). Constructing GSM in plants are also still fairly limited due to the complexity of their metabolic pathways and their interactions, complex regulation, and compartmentalization of metabolic processes (Rai et al., 2017). Furthermore, the modeling of secondary metabolite pathways still requires extensive manual curation, compared to primary metabolism (de Oliveira Dal'Molin and Nielsen, 2013; de Oliveira Dal'Molin and Nielsen, 2018). Hence, new, comprehensive data sets with organellar specific information are necessary for GSM improvement and validation in plants (Fukushima et al., 2014).

Protein regulation and post-translational modifications, such as phosphorylation are also known to impact the coordination of various cellular and biochemical processes (Nukarinen et al., 2016). Yet, they are unpredictable from any metabolic reconstruction (Weckwerth et al., 2020), complicating comprehensive and functional GSM. Metabolic and phenotypic dynamism are also affected by environmental factors, which thus far are not deductively informed by the genomes alone (Weckwerth et al., 2020). Hence, a “PANOMICS” platform integrating not just the expression omics (transcriptomics, proteomics, and metabolomics) as described in this review, but also other large-scale studies including epigenomics, post-translational modification proteomics, and phenomics (Weckwerth et al., 2020) could reveal the underlying intricate molecular regulation in plants. The use of machine learning will certainly aid in integrating these highly diverse omics platforms. Evidently, machine learning has recently been used to distinguish genes responsible for specialized metabolism important for plant-environment interaction (Moore et al., 2019) as well as precision breeding for certain traits of interest (Weckwerth et al., 2020). The development of effective machine learning algorithms (**Table 3**), especially deep learning approaches such as artificial neural networks to iteratively correct constructed genome-scale models will undeniably become an immediate future direction in plant MOI research (Rai et al., 2019; Silva et al., 2019; Weckwerth et al., 2020).

While multi-omics efforts can be highly applicable and useful in plant research, analyzing large-scale data sets can be a major bottleneck and it is hoped that updated reviews such as this can set as exemplary and methodological guidelines. Furthermore, multi-omics experimentation and integration in plants often require the right composition of research teams, even multi-nationally (Zivy et al., 2015), due to the organism complexity as discussed earlier. One such effort in promoting strong collaborative work is the COST Action FA1306 initiative, which aims to develop an effective workflow for diverse omics experimentation in various applications, including breeding and agriculture management (Zivy et al., 2015). More coordinated omics research works such as this could facilitate a comprehensive characterization from many more valuable plant species in various parts of the world.

## Conclusion

Advancement in omics technologies have generated a massive amount of data, thus an effective, methodological approach in data integration is needed to make sense and relate the data back to the objective of the research. This review suggests three levels of MOI approaches (levels 1, 2, and 3) ranging from simple element-based integration (correlation, clustering, and multivariate) and pathway-based approaches (pathway mapping and co-expression) to the more complex integration using mathematical approach (differential and genome-scale). These approaches were explained in view of current literature to highlight their applications and practicality in plant MOI. However, the limitation of each approach needs to be considered before embarking on any MOI studies particularly for less characterized non-model plants. Future work in improving MOI strategies can be dedicated to complete gene and metabolite annotation for specific plant species, as well as developing user-friendly tools utilizing machine learning algorithms to allow accurate metabolic model reconstruction.

## Author Contributions

WA, MA, and IJ designed the framework of the manuscript. IJ wrote the first draft of the manuscript. JR wrote sections of the manuscript. MA, KA, NN, H-HG, and WA revised the manuscript critically. All authors contributed to manuscript revision, read, and approved the submitted version.

## Funding

This research was funded by the UKM Research University grant (DIP-2018-001 and GUP-2018-122) to WA. The work is also partly supported by NBDC Database Integration Program (MA), and NIG-JOINT grant 2019 (2A2019) (IJ and H-HG), Japan.

## Conflict of Interest

The authors declare that the research was conducted in the absence of any commercial or financial relationships that could be construed as a potential conflict of interest.
